# Multidisciplinarity Is Critical to Unlock the Full Potential of Modern Light Microscopy

**DOI:** 10.3389/fcell.2021.739015

**Published:** 2021-10-21

**Authors:** Michael Weber, Jan Huisken

**Affiliations:** Morgridge Institute for Research, Madison, WI, United States

**Keywords:** light microscopy, open science, multidisciplinary, biology, imaging, technology, access

## Light Microscopy Has Become Increasingly Capable

**FIGURE 1:**
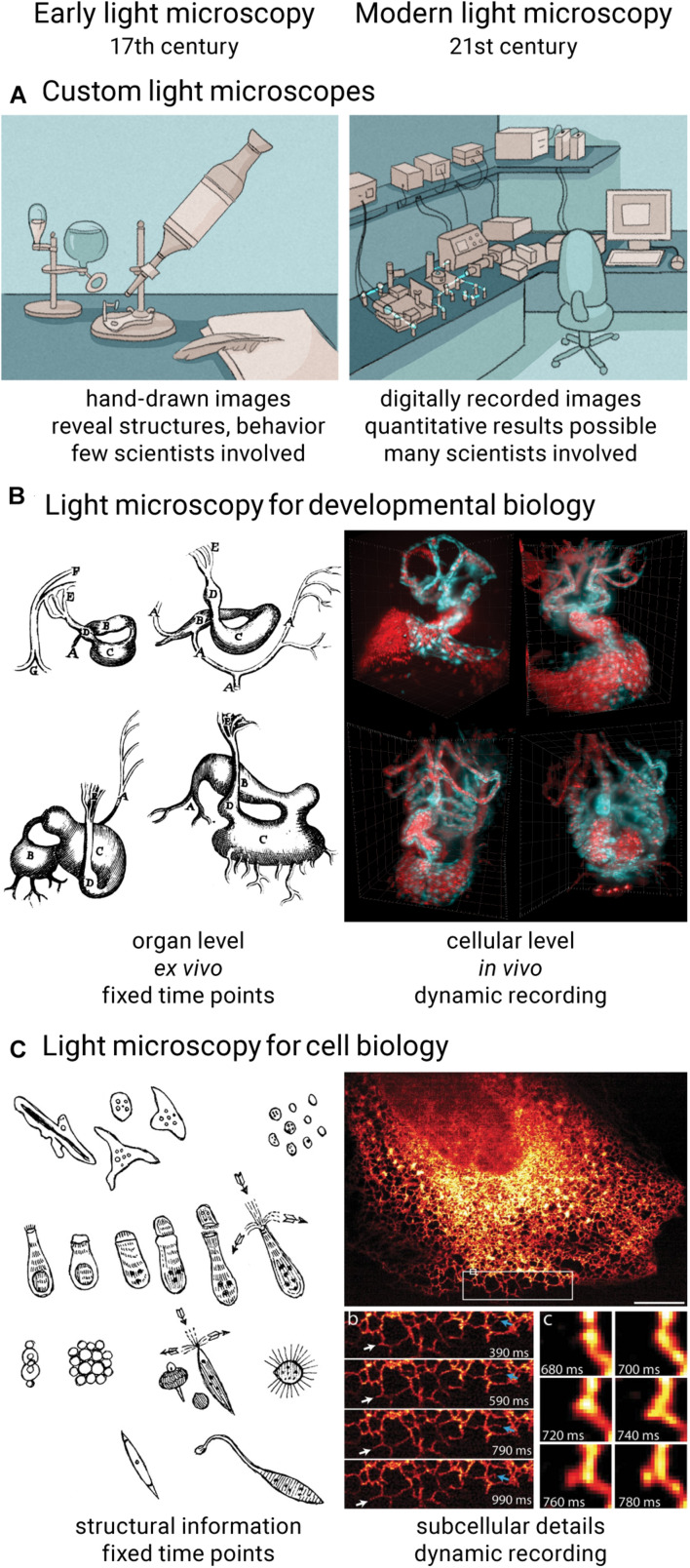
Comparison of early and modern light microscopy. Over the course of 400 years, light microscopes transformed from pure lens arrangements into complex and automated devices **(A)**. Light microscopy techniques in developmental biology can now capture dynamic processes in tissues, organs and organisms under nearly physiological conditions **(B)** (West, 2013; Mickoleit et al., 2014). In cell biology, light microscopes are now capable of recording fine subcellular details **(C)** (York et al., 2013; Rob et al., 2016). Previously published images reused under license 5144871109849.

Optical microscopy is a cornerstone of the biological sciences. It has become the most important imaging technique in biomedical research by providing high spatial resolution, high specificity, and suitability for living specimens. From the first microscopic observations of embryos and living cells in the 17th century over the first mass-produced optical microscopes and the formal definition of optical resolution in the 19th century to a sheer endless list of technological inventions that helped discover and unravel many biological mysteries throughout the 20th century, many scientifically minded people have contributed their part to develop and establish light microscopy as the powerful technique it is today (Rayleigh, 1896; Clara, 1966; van Zuylen, 1981; Paddock and Eliceiri, 2014; Zanacchi et al., 2014; Wollman et al., 2015; Maienschein, 2016). Despite its long history, light microscopy is experiencing rapid development in the 21st century. For example, recent developments like light sheet microscopy (Huisken et al., 2004) and super-resolution microscopy (Gustafsson, 2000; Klar et al., 2000; Rust et al., 2006; York et al., 2013) provide biologists with tools to image fragile organisms in close-to-native conditions over long periods of time (Figure 1B) and capture images of samples with spatial resolution exceeding the diffraction limit (Figure 1C), respectively.

## Modern Light Microscopy Presents Biologists With New Possibilities and Challenges

Technological advancements in light microscopy are driven by – and drive – biologists’ needs to study and explore their samples in more detail. Recently, there has been the desire to move from imaging single layers of cells to recording images in a more physiological context, e.g., three-dimensional cell cultures or whole organisms, to move more to live samples to avoid side effects of fixation, or to increase throughput and automation to gain statistically relevant results. As a result, modern light microscopes do not resemble the compact optical devices they once were and are now rather complex setups that blend well-proven lens arrangements with newly designed optics, powerful electronics and intelligent software (Figure 1A). Early light microscopy was driven by polymaths and involved only very few people: one person developing and building the microscope and a second person preparing samples and documenting microscopic discoveries (Clara, 1966; van Zuylen, 1981). Prior to the debut of film and later digital cameras in this field, microscopy images were drawn by hand (Morrison and Gardner, 2015). While good microscope performance was crucial to make new observations, the quality and usefulness of the images were largely determined by the biologist’s artistic skills and *a priori* knowledge applied in the process of drawing. Today’s digital imaging is crucial to record microscopic observations in a reproducible and quantifiable way, but an entirely new set of skills is required to be successful in this endeavor. Modern light microscopy has become a collaborative effort where many experts and a multitude of disciplines are needed to develop and use the technology to its full extent.

The desire to extract quantitative data from microscopy images and the increasingly multidisciplinary aspect of optical microscopy results in both opportunities and challenges. With many features of modern light microscopes, such as optical sectioning, reduced photo-damage, increased spatial and temporal resolution, multi-sample imaging, automation, or optical manipulation, biologists have the potential to gain exciting new insights into their sample of interest. Unfortunately, for many researchers these new features might not be accessible: the increasing technical complexity of light microscopy, the plethora of image data, and the multitude of skills needed challenge traditional biologists. For example, a lack of compatibility of existing microscope hard- and software asks for programming and engineering skills that are not taught in conventional biology courses. Moreover, rapid technological advancements and frequent scientific publications suggesting technological breakthroughs make it difficult to keep track of promising developments and judge their feasibility for specific imaging experiments. Many light microscopy techniques, such as stochastic optical reconstruction microscopy, structured illumination microscopy, deconvolution and multi-view microscopy, ask for post-processing steps like restoration, registration or reconstruction of hundreds or thousands of images before the final result is seen (Agard and Sedat, 1983; Gustafsson, 2000; Rust et al., 2006; Preibisch et al., 2010); the required computer skills and information technology infrastructure are rarely present in a biology lab.

## Commercial and Custom-Built Light Microscopes

From a biologist’s perspective, commercial microscope setups seem to provide all-in-one solutions to most of the aforementioned challenges. Indeed, more and more advanced imaging technology is a great opportunity for vendors to develop and promote well integrated light microscopes that balance consistent performance and ease of use. Such commercial setups can provide a list of benefits for many researchers and present them with the one accessible route to high-end optical microscopy. With a common user interface, good integration of established technologies, intelligent soft- and hardware solutions, more and more automation features and on-site support from the vendor, commercial microscopes can form the core of a biology lab’s imaging needs. However, commercial optical microscopes are often designed as “black boxes” with at least partly concealed hard- and software solutions to simplify the user experience and avoid user error, but also to protect the companies’ intellectual property. Together with a tighter system integration and images pre-processed with proprietary algorithms, such “turn-key” instruments might prevent researchers from custom-fitting their microscopes and integrating them in their individual imaging workflows. Last but not least, the adaptation of new optical microscope technologies in a lab environment is delayed by the time it takes the company to turn an invention into a stable, easy-to-use and serviceable product and make it commercially available, a process that typically takes many years. As a result, many new light microscopy techniques only become accessible to a wider user base in a streamlined fashion upon commercialization many years after the initial scientific publication.

For many light microscopy applications in the life sciences, the advantages of commercial setups easily outweigh their downsides. However, the biologists’ ingenuity and curiosity can quickly call for more tailor-made light microscopes, be it to pioneer a new sample or to test a new hypothesis with unconventional techniques. Those skilled in the art can build custom light microscopes around the sample with just the right combination of components, specifically tailored for novel biological imaging projects. Such custom-built microscopes tend to provide unique features and performance not available in commercial microscopes, such as physiological conditions for day-long imaging of Arabidopsis thaliana (Maizel et al., 2011) or high-speed microscopy and post-acquisition synchronization to reconstruct the beating zebrafish heart in three dimensions (Mickoleit et al., 2014). Because patents and company associations, as well as aspects like mass market compatibility, scalability and interface optimizations are of lower priority for scientists, custom microscopes can be finalized in a timely manner and provide quicker access to new technology. In many cases, though, the biologist’s desire for custom-built light microscopes is limited by a historical disconnect between scientific disciplines: optical microscopes are mostly developed in physics-oriented environments, often far away from biological samples and real-world applications. Therefore, biologists might not be aware of new developments and might not be able to access such microscopes. Even when published in full detail, custom microscopes can remain exclusive builds only accessible to the developer and close collaborators. Their often unique and complex designs require substantial engineering, optics, and computer science skills and make it next to impossible for interested biologists to build a similar setup, reproduce published results and facilitate custom microscope technology for their own imaging ideas.

## Open Science Is Gaining Importance

How well a technology is shared and used within a community is a good indicator of how collaborative its development has been. The inherent openness of multidisciplinary work directly contributes to more accessible, reliable, and reproducible science. In recent years, open science projects, such as the development of open-source soft- and hardware, gained more and more traction among developers and users of light microscopy. Today, biologists have access to open-source software for image analysis and even microscope control (Carpenter et al., 2006; Edelstein et al., 2010; Schindelin et al., 2012). Open hardware projects provide building plans for custom components to upgrade existing microscopes or build entire light microscope setups (Pitrone et al., 2013; Nicovich et al., 2017; Voigt et al., 2019; Diederich et al., 2020; Kumar et al., 2021). In addition, different models have been proposed to enable new biological experiments and give researchers better access to light microscopy technology by either streamlining the submission of fixed specimens or bringing mobile microscopes right to where the biological sample is located (Power and Huisken, 2019; Schweigreiter et al., 2019). While open solutions tend to require significantly lower initial costs, they might pose risks like unforeseeable additional investments and a lack of support. Here, institutions and funding agencies are asked to step in and provide reliable and long-term backing.

## Mastering Modern Light Microscopy Requires a Unique Combination of Skills

**FIGURE 2:**
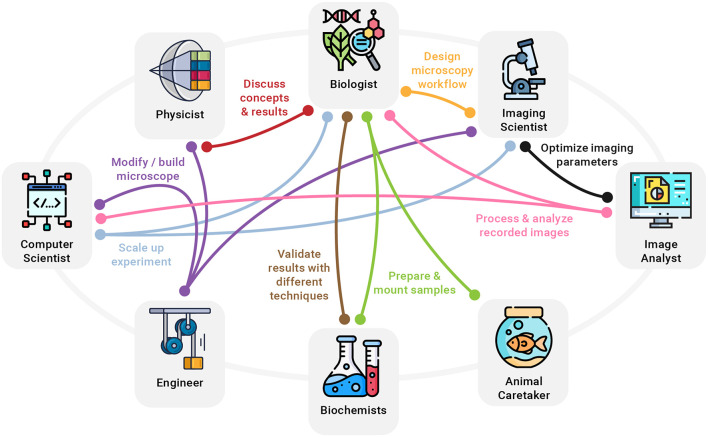
A few examples of how a multitude of disciplines need to work hand in hand to turn new imaging ideas into reality. For example, an imaging scientist can provide valuable help to the biologist in designing a clear and reproducible microscopy workflow, while a biochemist might be needed to develop specialized fluorophores. At the same time, computer scientists, physicists, and engineers collaborate to modify or build an optical microscope needed for the experiment.

In addition to the hurdles when accessing modern microscopy technology, making full use of it is not without challenges, either. Performing reproducible and scientifically sound imaging experiments is a complex task often only covered in passing during biology training (Boehm et al., 2021). Advertisements for commercial light microscopes can give the impression that such setups are straightforward to use, like a camera or a smartphone set to “Auto.” Along the same lines, scientific publications tend to oversell new microscopes and glance over shortcomings and difficulties when building, aligning, and using the technique on a regular basis. In reality, modern light microscopy, much like any other scientific technique, requires a diverse set of skills, good planning, time, and precise execution in order to yield reliable results. A lack of knowledge and not following established best practices can inadvertently lead to misleading conclusions (Lambert and Waters, 2017; Montero Llopis et al., 2021). Consequently, previously unrelated disciplines need to be included in bioimaging workflows to extend the skill set and make full use of new optical microscopy technologies emerging for biological research. Experts who can make meaningful contributions to common modern imaging experiments with biologists include physicists, engineers, biochemists, computer scientists, imaging scientists, image analysts, and animal caretakers, among others (Figure 2).

A large variety of disciplines covering everything that is needed to perform the ideal imaging experiment is rarely found in traditional biology labs – often not even within individual research institutions. Meanwhile, modern science has become a multidisciplinary effort, requiring diverse groups of researchers from different fields to work together to tackle scientific challenges. In fact, technology development in light microscopy does repeatedly benefit from multidisciplinary collaborations and from knowledge transfer across disciplines. Examples include the use of deconvolution to computationally reverse optical distortion, a signal processing technology that was first established in seismology and later applied in astronomy before a biochemist and biophysicist introduced it to optical microscopy (Wiener, 1949; Agard, 1984; Wallace et al., 2001). Another precedent is the development of photoactivated localization microscopy (PALM), a super-resolution technique that required scientists with different backgrounds to join forces and leverage knowledge of biology, biochemistry, optics, and image processing (Betzig et al., 2006; Hess and Betzig, 2010).

Importantly, multidisciplinary research helps form a more complete and objective description by combining different perspectives of the same scientific topic (Amato et al., 2019). For example, in an imaging experiment, a biologist might interpret varying intensities across one or more recorded images as different expression levels, whereas an imaging scientist might find it being caused by uneven illumination or an unstable light source (Figure 2). In addition, multidisciplinary work is invaluable for both well-designed presentations that communicate results to a broad audience and for better reporting on imaging methods for reproducible science (Marques et al., 2020). Future challenges in light microscopy will increasingly ask for close collaborations of different professions and the integration of insights from other scientific fields. For example, the growing need to integrate image processing and analysis into imaging workflows, as well as the increasing data sizes will further raise the importance of computer science. Furthermore, streamlining the design of microscope hardware and increasing its accessibility will require efforts from engineering. Last but not least, designing reproducible imaging experiments, encouraging open microscopy efforts, and establishing comprehensive light microscopy education will ask for more input from imaging scientists.

## Paths to More Multidisciplinarity

An individual research lab can become more multidisciplinary in two ways: establishing collaborations with labs of other disciplines or including researchers of different backgrounds in their own lab. A common example of the former is a collaboration between biology labs, microscope developers, and computer science labs, such as the one resulting in the first lattice light sheet microscope (Chen et al., 2014). In many ways, this is a fast way of extending and combining skill sets to successfully work on larger projects, but obtaining conclusive and reproducible results can take a lot of time and effort with every new collaboration. Whereas established structures of individual labs work well for internal projects, workflows and routines will need to be adjusted for effective external collaborations. In addition, good communication needs to be established between the labs, experts need to adjust their language and explain concepts specific to their discipline in more detail, and everyone involved must provide useful and understandable feedback to facilitate progress. Additional technical challenges include how biological samples, tools, and large amounts of data can be shared across labs and institutions. The second strategy, including most of the required disciplines in a single lab, is a long-term effort, but each imaging project will benefit and show results much faster. Multidisciplinary research labs with expertise from multiple fields interacting on a daily basis are at the forefront of developing viable light microscopy technology. Our own lab has embraced the idea of multidisciplinarity early on and has a long history of developing custom light sheet microscopy around specific biological samples and questions (Schmid et al., 2013; Weber et al., 2017; Daetwyler et al., 2019). These projects were team efforts from scientists of diverse disciplines collaborating in the lab every day and resulted in powerful microscopes that record the best possible images from the respective living and developing organisms. We are convinced that an optics development lab greatly benefits from including biologists, as it helps to connect with the biological community and steer microscope development in a meaningful direction. In the same way, we believe it is highly beneficial for a biology lab to hire scientists with different and often considered unrelated professions, such as engineering and computer science. Having all experts right in the lab is the most direct and effective way of establishing a multidisciplinary lab. All experts learn to speak the same language, share their experience and technology and expand their perspective every day, right from the start.

## Scientific Environments Need to Actively Support Multidisciplinary Work

Establishing a multidisciplinary lab requires not only a critical mass of people, but also a supportive institution. Especially in the early phase of building a research lab, its skill set can be extended by the right environment: animal and cell facilities can assist with handling and preparing samples, a machine shop can help out with tools and engineering skills, a computer department can build a reliable data backbone, and a light microscopy core facility can be a crucial contributor of imaging expertise (Ferrando-May et al., 2016; Lippens et al., 2019). Importantly, the support of the institution should go well beyond maintaining existing facilities and helping with technical challenges for collaborative efforts and multidisciplinary labs to succeed. Universities and research institutes need to accept and respect new lab structures and the inclusion of previously foreign disciplines and must see themselves as a unit of experts working together to do the best possible science, not as individual labs competing for independence. More appreciation from universities for unconventional projects and community efforts can lower the risks for individual labs and make additional investments for technology development worthwhile (Fantner and Oates, 2021). Research institutions also need to bolster core facilities with well-trained staff and allow for time and resources to engage into scientific collaborations and technology development (Adami et al., 2020; Ravindran, 2020; Waters, 2020). Ideally, the scientific environment not only accepts, but encourages scientists to engage in multidisciplinary work. Research institutions might find our list of core values (Box 1) helpful to make the productive, multi-disciplinary workplace a reality. As light microscopy keeps evolving over the coming years, multidisciplinarity on all levels will remain critical to unlock its full potential.

**Box 1. Core values research institutions should embrace to support collaborative and multidisciplinary work.**

1 Acknowledge multidisciplinarity as a strength and not dismiss it as being superficial and not specialized.

2 Appreciation of interdisciplinary work of individuals for career and publications.

3 Foster communication among experts of different disciplines.

4 Willingness to delve into new and unknown science.

5 Demand a proof of concept and a demonstration of meaningful applications beyond fundamental science.

6 Be critical of new ideas being sold as revolutionary when they are in fact only evolutionary.

7 Acknowledge the meaning of true innovation, which has to enable others to progress.

8 Expectation to share results and make them accessible for non-experts.

9 Work toward more open and reproducible science across all levels and disciplines.

## Data Availability Statement

The raw data supporting the conclusions of this article will be made available by the authors, without undue reservation.

## Author Contributions

MW wrote the first version of the manuscript. JH wrote sections of the manuscript. Both authors contributed to manuscript revision and read and approved the submitted version.

## Conflict of Interest

The authors declare that the research was conducted in the absence of any commercial or financial relationships that could be construed as a potential conflict of interest.

## Publisher’s Note

All claims expressed in this article are solely those of the authors and do not necessarily represent those of their affiliated organizations, or those of the publisher, the editors and the reviewers. Any product that may be evaluated in this article, or claim that may be made by its manufacturer, is not guaranteed or endorsed by the publisher.
